# Does Virulence Assessment of *Vibrio anguillarum* Using Sea Bass (*Dicentrarchus labrax*) Larvae Correspond with Genotypic and Phenotypic Characterization?

**DOI:** 10.1371/journal.pone.0070477

**Published:** 2013-08-06

**Authors:** Ingeborg Frans, Kristof Dierckens, Sam Crauwels, Ado Van Assche, Jørgen Leisner, Marianne H. Larsen, Chris W. Michiels, Kris A. Willems, Bart Lievens, Peter Bossier, Hans Rediers

**Affiliations:** 1 Laboratory for Process Microbial Ecology and Bioinspirational Management (PME&BIM), Thomas More Mechelen, Campus De Nayer, Department of Microbial and Molecular Systems (M2S), KU Leuven Association, Sint-Katelijne-Waver, Belgium; 2 Scientia Terrae Research Institute, Sint-Katelijne-Waver, Belgium; 3 Department of Veterinary Disease Biology, University of Copenhagen, Frederiksberg, Denmark; 4 Centre for Food and Microbial Technology, M2S, KU Leuven, Heverlee, Belgium; 5 Laboratory of Aquaculture and Artemia Reference Center, Department of Animal Production, Ghent University, Gent, Belgium; Beijing Institute of Microbiology and Epidemiology, China

## Abstract

**Background:**

Vibriosis is one of the most ubiquitous fish diseases caused by bacteria belonging to the genus *Vibrio* such as *Vibrio (Listonella) anguillarum*. Despite a lot of research efforts, the virulence factors and mechanism of *V. anguillarum* are still insufficiently known, in part because of the lack of standardized virulence assays.

**Methodology/Principal Findings:**

We investigated and compared the virulence of 15 *V. anguillarum* strains obtained from different hosts or non-host niches using a standardized gnotobiotic bioassay with European sea bass (*Dicentrarchus* labrax L.) larvae as model hosts. In addition, to assess potential relationships between virulence and genotypic and phenotypic characteristics, the strains were characterized by random amplified polymorphic DNA (RAPD) and repetitive extragenic palindromic PCR (rep-PCR) analyses, as well as by phenotypic analyses using Biolog’s Phenotype MicroArray™ technology and some virulence factor assays.

**Conclusions/Significance:**

Virulence testing revealed ten virulent and five avirulent strains. While some relation could be established between serotype, genotype and phenotype, no relation was found between virulence and genotypic or phenotypic characteristics, illustrating the complexity of *V. anguillarum* virulence. Moreover, the standardized gnotobiotic system used in this study has proven its strength as a model to assess and compare the virulence of different *V. anguillarum* strains *in vivo*. In this way, the bioassay contributes to the study of mechanisms underlying virulence in *V. anguillarum*.

## Introduction

Over the last five decades, the world aquaculture industry has grown considerably and according to the Food and Agriculture Organization (FAO), aquaculture represents the fastest-growing animal-food-producing sector [1]. This development has been accompanied by a transition to more intensive farming methods supporting an increased profitability. A side-effect of these intensified production systems is an increased threat by diseases caused by a variety of microorganisms, including bacteria, fungi, viruses and protozoa. Despite all research activity that has been carried out in order to develop rapid diagnostic tests and effective disease prevention strategies [2], large-scale disease outbreaks are still causing considerable economical losses [1].

Vibriosis, caused by *Vibrio (Listonella) anguillarum*, has been reported as one of the most important infectious diseases affecting many economically important fish, bivalves and crustaceans [3]–[5]. Symptoms are red spots on the ventral and lateral areas of the fish and swollen and dark skin lesions that can ulcerate and bleed. Furthermore, the eyes are often infected, initially resulting in opacity, and later on in ulceration and exophthalmia. Internally, the intestines may be distended and filled with a clear, viscous liquid. Outbreaks of this disease often result in high mortality rates of infected fish. Moreover, in acute epizootics, infection spreads so rapidly that the majority of infected fish die without showing any clinical signs [5]–[7]. Because of this high morbidity and mortality rate, the disease is responsible for severe economic losses in both larviculture and aquaculture worldwide. Disease outbreaks can be influenced by water quality and temperature, the amount of stress imposed upon the fish, and the strain and virulence of the bacteria. Out of 23 identified O-serotypes (O1–O23), only serotypes O1 and O2, and to a lesser extent serotype O3, have been linked to vibriosis in fish [8]. The other *V. anguillarum* serotypes represent environmental isolates from sediment, plankton or seawater that are mainly non-pathogenic. Over the last couple of years, knowledge of the virulence of this bacterium has been largely increased by the use of molecular and biochemical analysis approaches [9], [10]. In general, virulence factors in *V. anguillarum* have been classified into those that are necessary for chemotaxis and motility, for adhesion and invasion, including proteases [11], [12], hemolysins [13]–[16], lipopolysaccharides (LPS) [17]–[19], and those that are required for bacterial proliferation and persistence. Regarding the latter, it is for example known that siderophore-mediated iron-sequestering systems, enabling growth in iron-limiting conditions, contribute significantly to the virulence of this pathogen. Two different siderophore-mediated systems have been described in *V. anguillarum* strains. In most pathogenic strains of serotype O1, the system is mediated by the 65 kb virulence plasmid pJM1, harbouring genes for the biosynthesis of the siderophore anguibactin and its cognate transport system [20]. In contrast, serotype O2 strains and some plasmidless serotype O1 strains produce a chromosomally encoded siderophore vanchrobactin [21]–[24]. Nevertheless, despite these studies, the exact role and contribution of many of these virulence factors in full *V. anguillarum* virulence remain still largely unknown, e.g. by the lack of epidemiological studies and standardized virulence assays.

Recently, a gnotobiotic sea bass (*Dicentrarchus labrax*) larvae model system has been developed and optimized for studying host-pathogen interactions and virulence assessment of opportunistic pathogens such as *Aeromonas hydrophila* and *V. anguillarum* [25]. The European sea bass was chosen as host because of its high economic importance for larviculture and aquaculture [26]. Briefly, in this system, gnotobiotic larvae are challenged via immersion in inoculated water or encapsulated *Artemia* sp., followed by an assessment of the survival of the sea bass larvae. In this paper, we investigated and compared the virulence of 15 *V. anguillarum* strains obtained from different hosts or non-host niches using this standardized gnotobiotic bioassay. Here, the assay has been used for the first time to evaluate the virulence of a comprehensive set of different *V. anguillarum* strains. In addition, to assess potential relationships between virulence and genotypic and phenotypic characteristics, the strains were characterized by two complementary PCR-based genotyping methods, including repetitive extragenic palindromic PCR (rep-PCR) and random amplified polymorphic DNA (RAPD) PCR, and phenotypic analysis using Biolog’s Phenotype MicroArray™ technology [27].

## Materials and Methods

### Ethics Statement

All necessary permits were obtained for the described virulence studies. The experiment was approved by the ethical committee of Ghent University (no. EC2005/95).

### Bacterial Strains

Fifteen *V. anguillarum* strains, representing the major pathogenic serotypes O1, O2 and O3, were used in this study (Table 1). Fourteen strains have been isolated from different species of fish, including sea bass, rainbow trout (*Oncorhynchus mykiss*) and cod (*Gadus morhua* L.), while one isolate was recovered from sediment. Furthermore, the isolates were originating from six geographical regions, including Denmark, Norway, Finland, Greece, France and UK. In previous studies, most isolates have been assessed for the presence or absence of the pJM1 virulence plasmid (Table 1) [8], [28]–[33]. Nevertheless, as bacterial strains may lose plasmids over time, presence of the virulence plasmid has been confirmed in this study by isolation and sequencing. In addition, bacterial identities were confirmed as *V. anguillarum* by 16S rRNA gene sequencing, followed by BLAST analysis against GenBank (see phylogenetic analysis). Strains were stored in trypticase soy broth (TSB; Oxoid, Erembodegem, Belgium) containing 1% NaCl and 15% (v/v) glycerol at −80°C.

**Table 1:** *Vibrio anguillarum* strains used in this study.

Strain	Host or environment	Origin	Serotype	Virulence plasmid^a^	Reference
VIB15	Sea bass	Greece	O1	+	28
VIB93	Rainbow trout	Denmark	O1	+	28
87-9-116	Rainbow trout	Finland	O1	−	30
87-9-117	Rainbow trout	Finland	O1	+	30
VaNT1	Rainbow trout	Denmark	O1	+	8
S3 4/9	Mucus, Rainbow trout	Denmark	O1	−	29
JLL237	Rainbow trout	Hjarnø, Denmark	O1	−	33
43	Sea bass	UK	O1	−	31
VIB12	Sea bass	Greece	O2	−	28
VIB103	Cod	Denmark	O2	−b	28
VIB160	Sediment	Denmark	O2	−	28
JLL143	Rainbow trout	Denmark	O2	−	33
HI610	Cod	Norway	O2	−	Parisvannet, Norway
VIB113	Rainbow trout	Denmark	O3	−	28
CNEVA NB11008	Sea bass	France	O3	−	32

a + = Present; − = Absent (also confirmed in this study).

b Presence of plasmid >200 kb.

### Virulence Testing

The 15 selected isolates were made rifampicin (Rif) resistant by inoculating the strains at a final density of 10^7^ CFU mL^−1^ in marine broth with addition of an increasing concentration (1–100 mg L^−1^) of Rif. After 24 hrs culturing on a shaker at 28°C, 1% of the old culture is used as inoculum for the next culture untill a resistance for 100 mg L^−1^ Rif by natural mutation is reached. Next, the 15 isolates were subjected to the virulence assay previously developed by Dierckens et al. [25], consisting of a standardized gnotobiotic model system with axenic European sea bass (*D. labrax* L.) larvae as hosts. To this end, *D. labrax* eggs were obtained from natural spawning at the hatchery of Ecloserie Marine de Gravelines (France). Following egg disinfection [25], germ-free eggs were allowed to hatch for 60 h. Subsequently, 12 freshly hatched sea bass larvae were aseptically transferred one by one into a transparent sterile screw cap vial with 10 mL filtered (0.2 µm), autoclaved sea water (FASW) containing 10 mg L^−1^ Rif and sterile fish homogenate equal to three dead sea bass larvae, providing some nutrients to the bacteria in a gnotobiotic environment. Vials were rotated at 4 rpm with an axis tangential to the axis of the vials, providing aeration and avoiding sedimentation awaiting bacterial inoculation. Five days after hatching (DAH 5), sea bass larvae were counted for the first time. In addition, bacterial strains were grown in 10% marine broth containing NaCl (resulting in the same salinity as the water in the sea bass larvae experiment, i.e. 36 g L^−1^), and incubated on a horizontal shaker at 120 rpm at 16±0,5°C for two days. On DAH 7, the gnotobiotic sea bass larvae were challenged with a suspension of approximately 10^5^ cfu mL^−1^ *V. anguillarum* (as determined by a spectrophotometer at 550 nm). On DAH 9, 11, and 13 (i.e. 2–6 days post-exposure), survival of the sea bass larvae was monitored by microscopic analysis, i.e. by counting the number of living sea bass larvae in relation to the situation at DAH 7. For each strain, 10 replicates were evaluated. Non-challenged larvae, kept under similar conditions, were used as a control. In order to verify the gnotobiotic status of the assay, axenity was tested on DAH 3 by plating fish larvae homogenates and water samples as described by Dierckens et al. [25]. No bacteria could be detected after 72 h incubation. In addition, after the larvae survival experiment, homogenized fish larvae were plated on 10% marine agar (MA; Difco Laboratories, Detroit, USA) to check for microbial contamination as well as to verify the identity of the inoculated strains by DNA analysis (RAPD fingerprinting; see further). Throughout the entire experiment, eggs and (challenged) larvae were kept at a salinity of 36 g L^−1^ in a temperature-controlled room at 16±0.5°C in constant dim light (10 candela steradian m^−2^). Statistical analysis of the larval survival data was performed by means of R v2.12.1. Survival was reported as mean values ± standard error of the mean (SEM). Data were tested for normality and subjected to non-parametric tests. Kruskal–Wallis one-way analysis was used to compare the survival of the sea bass larvae. Bonferroni test was used for multiple comparisons among means in case of non-homogeneity. Significance was accepted at p<0.05.

### Phylogenetic Analysis

Genomic DNA was extracted following overnight incubation on marine agar at 28°C, using the phenol/chloroform extraction method as described by Lievens et al. [34]. Subsequently, the 16S ribosomal RNA (rRNA) gene, as well as the genes encoding an N-acetylmuramoyl-L-alanine amidase involved in the separation of daughter cells after cell division (*amiB)* [35] and an extracellular metalloprotease involved in virulence in *V. anguillarum* (*empA)* [11] were partially amplified with the primers 63F and 1492R, ami8 and ami417, and empAF and empAR, respectively [36]–[39]. PCR amplification was performed in a total volume of 20 µl containing 0.3 µM of each primer, 0.3 mM of each deoxynucleoside triphosphate (Invitrogen, Merelbeke, Belgium), 2.0 U *Taq* DNA polymerase (Bioké, Leiden, The Netherlands), 10X ThermoPol Reaction Buffer (Bioké), and 1 ng genomic DNA (as measured by a Nanodrop spectrophotometer). Before amplification, DNA samples were denatured at 94°C for 2 min. Subsequently, 35 cycles of the following steps were run: 45 s at 94°C, 45 s at 59°C (16S rRNA gene, *empA*) or 54°C (*amiB*), and 45 s at 72°C, followed by a final extension step at 72°C for 10 min. Sequencing of purified PCR products was performed using the forward primer used for DNA amplification for the *empA* and *amiB* amplicons and using the forward and reverse primer for the 16S rRNA gene amplicons. For the latter, forward and reverse sequences were individually trimmed for quality based on the obtained electropherogram, using a Phred-score of >20 (i.e. 0.01% error rate) as a cut-off value. Paired sequences were then aligned using the ClustalW algorithm within the MEGA5 software package [40], followed by manual sequence editing based on the paired electropherograms, leading to an accurate consensus sequence. Subsequently, a phylogenetic analysis was performed based on the *in silico* concatenated nucleotide sequences comprising 16S rRNA, *amiB* and *empA*. To this end, following multiple sequence alignment performed using ClustalW a Maximum Likelihood tree was constructed using the MEGA5 software. The sequences obtained in this study were deposited in GenBank under the accession numbers KF150774 to KF150818.

### DNA Fingerprinting

DNA extracted from all isolates studied was subjected to two fingerprinting techniques, including RAPD and rep-PCR. With regard to the RAPD analysis, first 20 decamer oligonucleotides, randomly chosen from the Operon primer kits (Operon Technologies Inc, Alameda, CA, USA), were screened on a subset of five bacterial strains from Table 1 to select the most discriminative RAPD primers. Two primers, OPV-12 (5′-ACCCCCCACT-3′) and OPN-08 (5′-ACCTCAGCTC-3′), resulted in a clear and discriminating fingerprint, and these were selected for further experiments. Likewise, for the rep-PCR analysis, two primers and one primer set were first tested on the same set of five isolates, including the BOXA1R primer (5′-CTACGGCAAGGCGACGCTGACG-3′), the (GTG)5 primer (5′-GTGGTGGTGGTGGTG-3′) and the primer pair REP1R-I (5′-IIIICGICGICGICATCIGGC-3′) and REP2-I (5′-ICGICTTATCIGGCCTAC-3′) [41]. As the BOXA1R and (GTG)5 primers generated only very weak bands or an insufficient number of fragments, only REP1R-I and REP2-I, yielding a clear and discriminating fingerprint, was maintained for analysis of the whole collection. All amplifications were performed using a Bio-Rad T100 thermal cycler in a reaction volume of 20 µl containing 0.5 µM of each primer, 0.15 mM of each deoxynucleoside triphosphate (Invitrogen), 2.0 U *Taq* DNA polymerase (Bioké), 10X ThermoPol Reaction Buffer (Bioké), and 1 ng genomic DNA (as measured by a Nanodrop spectrophotometer). The reaction mixture was initially denatured at 94°C for 2 min, followed by 35 cycles of 1 min at 94°C, 1 min at 35°C (RAPD) or 40°C (rep-PCR), and 2 (RAPD) or 4 min (rep-PCR) at 72°C, with a final extension step at 72°C for 10 min. Obtained PCR products were separated by loading 7 µl of the reaction volume on a 1% (w/v) agarose gel followed by 120 min electrophoresis at 4 V/cm in 1×Tris/acetate-EDTA (TAE) buffer. Gels were stained with ethidium bromide and visualized with UV light. A 1 kb DNA ladder (Smartladder; Eurogentec, Seraing, Belgium) was used as molecular weight marker. Gel images were acquired with the BioChemi System (UVP, Upland, CA, USA). Obtained images were processed by using GelCompar software, version 6.6.4 (Applied Maths, Sint-Martens-Latem, Belgium). Following normalization and background subtraction, fingerprint similarities based on the combined dataset were calculated using the Pearson correlation coefficient. Cluster analysis was performed by the unweighted pair group method with arithmetic averages (UPGMA) [42]. All reactions were performed at least twice to check reproducibility, and yielded similar results. In all analyses, sterile distilled water was used as a negative control.

### Phenotyping

For each strain, carbon source oxidation was determined by Phenotype MicroArray™ (PM) technology (Biolog, Hayward, CA) using PM plate 1. Using this technology, kinetic profiles are generated by continuously monitoring the metabolic activity during incubation [27]. The inoculum was prepared by growing strains for 24 hours at 25°C on trypticase soy agar supplemented with 1% NaCl. Cells were suspended in 10 ml inoculation fluid (IF-0 supplemented with 1% (w/v) NaCl, Biolog) until an optical density (600 nm) of 0.38 (±0.02) was reached, using a SPECTRAmax PLUS384 UV-vis spectrophotometer (Molecular Devices). The inoculum was diluted (1∶5) in inoculation fluid containing dye mix D (Biolog). Each well was inoculated with 100 µl. Plates were incubated in the OmniLog® automated incubator-reader of Biolog for eight days at 25°C and were read every 15 minutes. Resulting data were analyzed using OmniLog® PM Kinetic Analysis software (version 1.6) according to the manufacturer’s instructions. Comparison of the isolates was performed by numerical analysis using the Pearson product–moment correlation coefficient and hierarchical clustering with UPGMA. The clustering results were validated using cophenetic correlation [43]. A selection of reactions was performed at least twice to check reproducibility, and yielded similar results.

Additional phenotypic assays associated with virulence have been performed. All enzymatic and hemolytic assays were done according to Natrah et al. [44]. Briefly, overnight cultures of each bacterial strain were diluted to an OD600 of 0.5. For each assay, 5 µl of diluted culture was spotted in the middle of the test plate. MA plates supplemented with 1% Tween 80 (Sigma–Aldrich) or 1% egg yolk emulsion (Sigma–Aldrich) were used for the lipase and phospholipase assays, respectively. The development of opalescent zones around the colonies was observed and the diameter of the zones was measured after 2–4 days of incubation at 28°C. The caseinase assay plates were prepared by mixing double strength MA with a 4% skim milk powder suspension (Oxoid), sterilized separately at 121°C for 5 min. Clearing zones surrounding the bacterial colonies were measured after 2 days of incubation. Gelatinase assay plates were prepared by mixing 0.5% gelatin (Sigma–Aldrich) into MA. After 7 days of incubation, saturated ammonium sulfate (80%) in distilled water was poured over the plates and after 2 min, the diameters of the clearing zones around the colonies were measured. Hemolytic assay plates were prepared by supplementing MA with 5% defibrinated sheep blood (Oxoid). Clearing zones were measured after 2 days of incubation. All assays were done at least in triplicate.

## Results

### Virulence Testing

**Figure 1:**
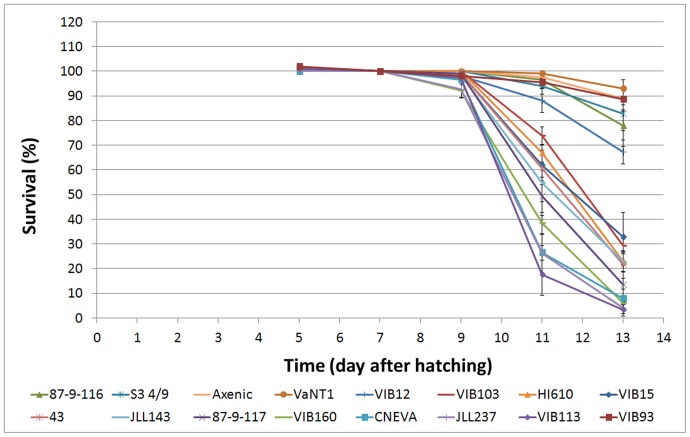
Survival (in percentage) of sea bass larvae during the gnotobiotic experiment (unfed larvae) before and after challenge (7 days after hatching) with the 15 selected *Vibrio anguillarum* isolates. Error bars represent mean ± SEM (n = 10).

In order to assess the virulence of 15 *V. anguillarum* isolates, gnotobiotic sea bass larvae were challenged with *V. anguillarum*, whereupon the larval survival was monitored by microscopical analysis at DAH 9, 11 and 13 (Fig. 1; Table 2). After DAH 13 the sea bass larvae in the control treatment died of starvation, making us to decide to terminate the experiment at DAH 13 (six days after inoculation). Survival on DAH 13 of the sea bass larvae challenged with the serotype O1 strains VaNT1, VIB93, S3 4/9 and 87-9-116 (ranging from 93±3% to 78±8% survival) was not significantly different from the control group (89±4%). Treatment with the O2 strain VIB12 (67±5%) was significantly different from the axenic control but not significantly different from inoculations with S3 4/9 and 87-9-116. As such, based on these observations these five strains were classified as avirulent strains, at least in the gnotobiotic system used here. The other ten strains caused a significantly higher mortality compared to the avirulent strains as well as the axenic control and were classified as virulent strains. Strains VIB15 and 87-9-117, two O1 serotype strains containing the virulence plasmid, showed high virulence towards sea bass larvae (33±10% and 13±8% survival, respectively). Remarkably, although strain 87-9-117 was originally isolated from rainbow trout, it also caused high mortality in sea bass larvae, indicating that there is no stringent host-specificity for vibriosis. Two other O1 serotype strains (43 and JLL237), although lacking the virulence plasmid, were also virulent (22±5% and 4±2% survival, respectively). Strains VIB103, JLL143, and VIB160, belonging to serotype O2 also caused high mortality (29±3%, 23±4%, and 6±2% survival, respectively). Apparently, although strain VIB160 was not isolated from fish but from sediment, it showed high virulence towards sea bass larvae. In addition, all tested O3 strains (VIB113 and CNEVA NB11008) appeared to be highly virulent (3±3% and 8±4% survival, respectively). An independent replication of this gnotobiotic sea bass challenging experiment confirmed the results shown in Fig. 1. For each experiment, the identity of the inoculated strains was confirmed by isolating the bacteria again at the end of the experiment (DAH 13) followed by RAPD fingerprinting, confirming that mortality was caused by the tested *V. anguillarum* strains and not by non-added strains.

**Table 2:** Genotypic and Phenotypic properties of the *Vibrio anguillarum* strains used in this study.

Strain	Serotype	Virulence assay(% survival)^a^	Cluster Genotyping^b^	Cluster Phenotyping^c^	Enzyme assays^d^				
					Lipase	Phospholipase	Caseinase	Hemolytic	Gelatinase
VIB15	O1	33±10	I	A	−	+	+	+	+
VIB93	O1	89±5	I	A	+	+	+	+	+
87-9-116	O1	78±8	I	A	+	+	+	−	+
87-9-117	O1	13±8	I	A	+	+	+	+	+
VaNT1	O1	93±3	I	A	+	+	+	+	+
S3 4/9	O1	83±7	II	B	+	+	+	+	+
JLL237	O1	4±2	IV	B	+	+	+	+	+
43	O1	22±5	V	C	+	+	+	−	+
VIB12	O2	67±5	II	E	+	+	−	−	+
VIB103	O2	29±3	V	B	+	−	+	+	+
VIB160	O2	6±2	II	B	+	+	+	+	+
JLL143	O2	23±4	III	D	+	+	+	+	+
HI610	O2	23±4	V	C	+	−	+	+	+
VIB113	O3	3±3	II	D	+	+	+	+	+
CNEVANB11008	O3	8±4	II	B	+	+	+	−	+

a Results from DAH 13, the mean % survival ± SEM is presented (n = 10) (see also Fig. 1).

b Clustering results (combined datasets) at a similarity level of 50% (see also Fig. 3).

c Clustering results at a similarity level of 90% (see also Fig. 4).

d Enzyme activity:+ = Present; − = Absent.

### DNA Analysis

**Figure 2:**
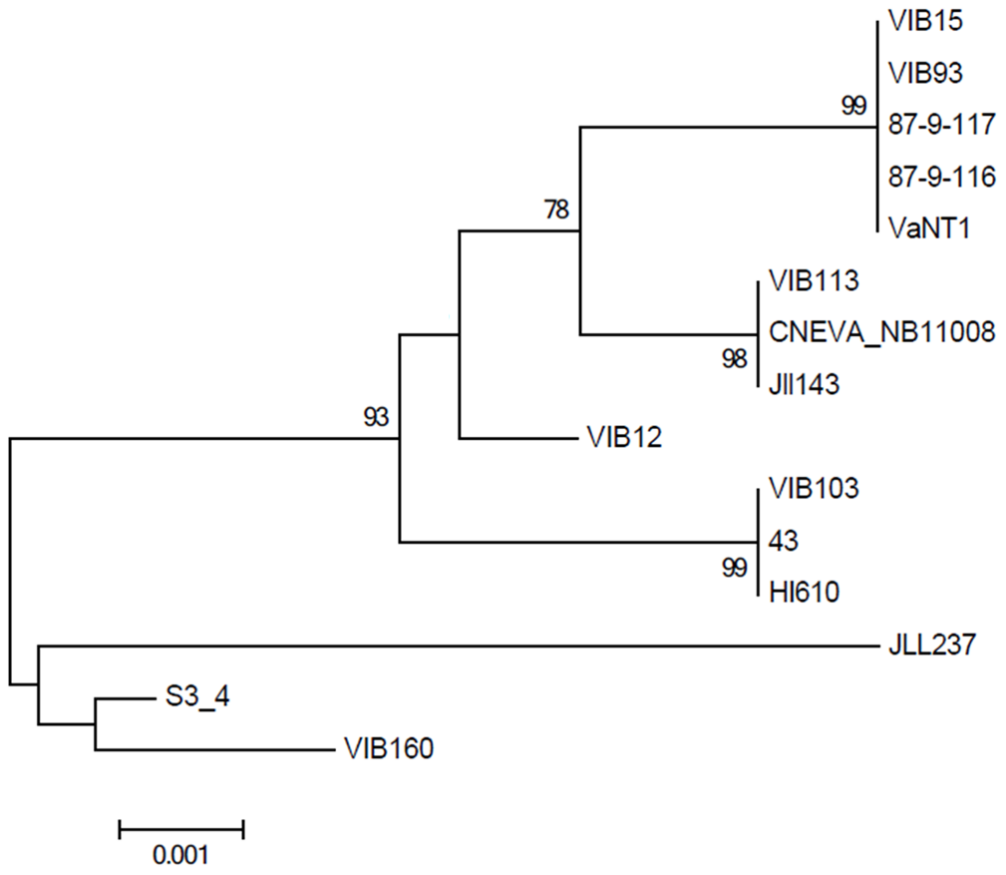
Maximum Likelihood phylogram based on a 2101 bp concatamer of 16S rRNA, *amiB* and *empA* gene sequences of the *Vibrio anguillarum* isolates investigated in this study. The percentage of trees from 1000 bootstrap resamples supporting the topology is indicated when above 50.

To assess a potential relation between virulence and genotypic background, all strains were subjected to both a phylogenetic analysis and DNA fingerprinting. First, a phylogenetic tree was constructed based on the *in silico* concatenated sequences of the 16S rRNA gene (1365 bp), *amiB* (365 bp) and *empA* (371 bp) sequences (Fig. 2). However, due to the high degree of conservation of these genes (98–100% similarity), not all strains could be discriminated from each other. In particular, five out of the eight O1 strains (87-9-116, 87-9-117, VaNT1, VIB93 and VIB15) showed identical 16S rRNA, *amiB* and *empA* sequences, as did the two O3 strains (VIB113, CNEVA NB11008) (Fig. 2).

**Figure 3:**
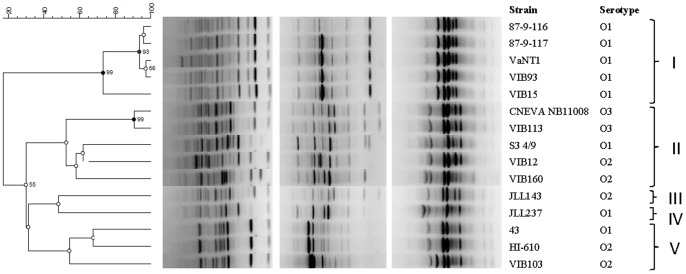
RAPD (two gels on the left) and rep-PCR (gel on the right) fingerprints and corresponding dendrogram (combined datasets) derived from UPGMA linkage of Pearson correlation coefficients of the *Vibrio anguillarum* isolates investigated in this study. The percentage of trees from 100 bootstrap resamples supporting the topology is indicated when above 50.

Next, two RAPD analyses (primers OPV-12 and OPN-08) and one rep-PCR analysis (primer pair REP1R-I and REP2-I) were performed. RAPD and rep-PCR patterns were obtained yielding 5 to 14 distinct bands. The UPGMA dendrogram derived from Pearson correlation based on the combined datasets showed high congruence with the phylogenetic tree (Fig. 3; Table 2). However, the discriminative power displayed was considerably higher with the fingerprinting methods. Only strain VIB12, belonging to serotype O2, clustered differently in both trees. Based on a similarity level of 50%, 5 distinct clusters could be identified in the UPGMA dendrogram. Cluster I only contained O1 serotype strains (5 out of the eight studied), while cluster II was comprised of the two O3 strains (fingerprints sharing 100% similarity), two O2 serotype strains (VIB12 and VIB160) and one O1 strain (S3 4/9) as well. Cluster V is represented by two O2 strains (HI610 and VIB103), supplemented with one O1 strain (43). Isolates JLL237 (O1 serotype) and JLL143 (O2 serotype) landed in a separate cluster, cluster III and IV, respectively (Fig. 3). Although there is some correspondence between genetic clustering and serotype, our results do not suggest a correlation with the results obtained in our virulence assay, as both the avirulent and virulent strains appear scattered throughout the dendrogram.

### Phenotypic Characterization

For all *V. anguillarum* strains, high metabolic activity was observed, except for the O2 strain VIB12, which only consumed 12 carbon sources (L-arabinose, 2′-deoxyadenosine, adenosine, D-glucose-1-phosphate, D-glucose-6-phosphate, D-fructose-6-phospate, L-lyxose, pyruvic acid, inosine, fumaric acid, L-asparagine, and D-ribose). These findings were confirmed in an independent experiment. Out of 95 carbon sources tested in this study, 40 were consumed by all other 14 *V. anguillarum* strains (L-arabinose, D-xylose, acetic acid, L-aspartic acid, L-lactic acid, L-alanine, 2′-deoxyadenosine, adenosine, D-glucose-6-phosphate, D,L-malic acid, D-fructose-6-phospate, succinic acid, citric acid, L-lyxose, uridine, pyruvic acid, D-sorbitol, inosine, D-gluconic acid, L-malic acid, L-serine, fumaric acid, β-methyl-D-glucoside, L-asparagine, glycerol, Ala-Gly, L-threonine, Gly-Pro, L-glutamic acid, D-alanine, D-fructose, D-mannose, α-D-glucose, sucrose, D-mannitol, L-glutamine, L-proline, D-galactose, D-ribose, and N-acetyl-D-glucosamine), while 20 were only metabolized by some strains and 35 by none of the strains tested (D-glucuronic acid, D-glucosaminic acid, 1,2-propanediol, α-methyl-D-galactoside, L-rhamnose, D-threonine, mucic acid, D-aspartic acid, L-fucose, a-hydroxyglutaric acid-g-lactone, D-galactonic acid-γ-lactone, D-saccharic acid, m-tartaric acid, adonitol, glyoxylic acid, glycolic acid, α-D-lactose, N-acetyl-D-mannosamine, acetoacetic acid, lactulose, tricarballylic acid, tyramine, glucoronamide, D-melibiose, m-hydroxyphenyl acetic acid, D-malic acid, L-galactonic acid-γ-lactone, mono-methylsuccinate, p-hydroxyphenyl acetic acid, D-psicose, dulcitol, thymidine, 2-aminoethanol, D-galacturonic acid, and phenylethylamine) (Table S1).

**Figure 4:**
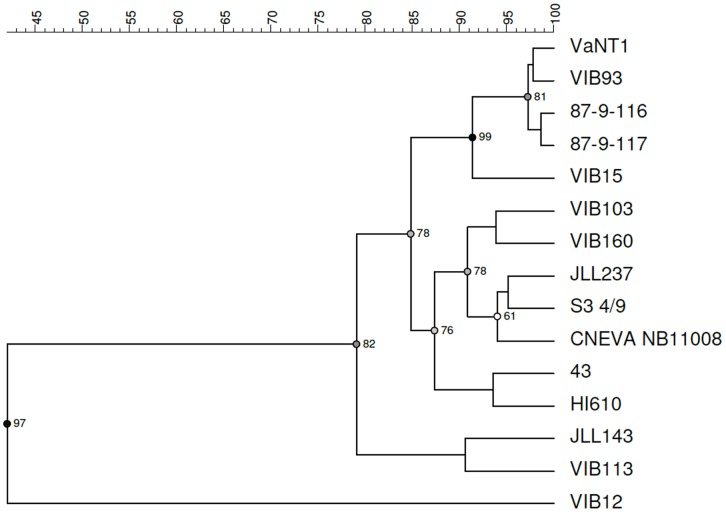
Biolog PM1 phenotypes within *Vibrio anguillarum* strains. The dendrogram was derived by UPGMA cluster analysis of the Biolog PM1 data (i.e. area under the curve) of the 15 selected *V. anguillarum* strains. The results were validated using cophenetic correlation.

Next, the *V. anguillarum* strains were clustered based on the ability to utilize different carbon sources. As can be seen from the UPGMA dendrogram constructed from the PM1 profiles (area under the curve), clear differences can be observed between the studied *V. anguillarum* strains (Fig. 4; Table 2). Based on 90% similarity, five clusters can be identified, of which cluster A perfectly matched cluster I obtained by genotyping. The two O3 strains, CNEVA NB11008 and VIB113, which clustered together by genotyping (cluster II), were separated in this analysis (cluster B and D, respectively). Strains HI610 (O2 serotype) and 43 (O1 serotype) clustered together in cluster C, which is in agreement with the genotyping results (clusters V). Cluster B contained two O1 strains, JLL237 and S3 4/9, two O2 strains, VIB103 and VIB160, and one O3 strain, CNEVA NB11008. Cluster C contained strains JLL143 (O2 serotype) and VIB113 (O3 serotype). As previously mentioned, the O2 strain VIB12 showed very low metabolic activity and therefore clustered separately. Similar to the genotypic analysis, no correlation could be observed between virulence and the phenotypic analysis.

In addition, the production of extracellular enzymes, which are possibly involved in virulence and invasion of host tissue [44], of the 15 *V. anguillarum* strains was determined on agar plates containing various substrates (Table 2). Strain VIB12 lacked both caseinase and hemolytic activity, which may explain its avirulent behavior. Remarkably, although strain VIB15 was identified as a virulent strain towards sea bass larvae, it was the only strain which did not contain lipase activity. Likewise, two virulent O2 strains, VIB103 and HI610, were deficient in phospholipase activity. Finally, no hemolytic activity was detected in the avirulent strain 87-9-116, and the virulent strains 43 and CNEVA NB11008, belonging to serotype O1, O2 and O3, respectively (data not shown).

## Discussion

In this study, a set of 15 *V. anguillarum* strains from different origins (sea bass, trout and cod, or sediment) were subjected to a previously designed standardized virulence assay using axenic European sea bass larvae as hosts [25]. Here, the utility of the method to assess and compare the virulence of different *V. anguillarum* strains was demonstrated on a larger scale for the first time. Out of the 15 tested strains ten isolates were categorized as virulent strains, whereas five isolates were found to be avirulent. In general, our results were in line with other virulence characterization studies of *V. anguillarum*. For example, strains VIB15, VIB103, 87-9-117, JLL143, JLL237 and CNEVA NB11008, which were found to be virulent in other studies ([28], [32], [33], M. Halberg Larsen, unpublished data), were also virulent in our study. Additionally, strains VIB93, 87-9-116, VaNT1 and S3 4/9 which were avirulent in earlier studies ([8], [28], [30], [45], M. Halberg Larsen, unpublished data), were avirulent here as well. On the other hand, the virulence characteristics of strains VIB160 and VIB113, which were both virulent in our study, were in contrast to previous studies [28]. This discrepancy may be explained by a different host specificity combined with the use of a different infection model system, i.e. intraperitoneal injection of Atlantic salmon versus challenge of sea bass larvae by immersion [46], [47]. Additionally, despite strain VIB12 was originally isolated from a diseased sea bass, this strain was found to be avirulent in our screening system. Potentially, this could be explained by the developmental stage of the fish or potential co-infections facilitating entry of *V. anguillarum* in the adult fish.

Although the virulence plasmid pJM1 is generally regarded as an important virulence factor in *V. anguillarum*, no strict relation between the presence of the plasmid and virulence was detected in our study. For example, serotype O1 strains VaNT1 and VIB93, both containing the virulence plasmid [8], [28], were found to be avirulent in our study. It is well known that bacteria can be deprived of their plasmids during storage or passage of the strains [48]. Therefore, presence or absence of pJM1 was verified for all our strains based on plasmid isolation and sequencing, confirming the strain characteristics. The avirulent character of these strains on sea bass larvae, both originally isolated from rainbow trout, may be explained by a stringent host specificity, which may differ from strain to strain (see earlier). In addition, the lack of virulence of VaNT1 may be explained by its “rough” colony morphology [8]. It has been recognized that rough strains, which lack the lipopolysaccharide O-antigen, are more susceptible to complement-mediated killing, resulting in an avirulent phenotype [17], [49]. In contrast, two other O1 serotype strains that contain the virulence plasmid (VIB15 and 87-9-117) did result in a high mortality of the sea bass larvae, and, remarkably, strains lacking the virulence plasmid were found to cause high mortality in our study system (43 and JLL237). However, as plasmidless O1 strains may have a chromosome-encoded iron sequestering system instead of the pJM1 plasmid-encoded iron uptake system, its function may have remained intact, contributing to its virulence [50], [51]. These data strongly suggest that presence of the virulence plasmid is not crucial for full virulence towards sea bass larvae.

In order to assess potential relationships between virulence and genotypic and phenotypic characteristics, the strains were subjected to both genotypic and phenotypic characterization. Next to sequence analysis of a number of housekeeping genes, strains were genotyped using RAPD and rep-PCR, both used successfully for epidemiological studies of fish pathogens previously [52]–[55]. The clustering based on the genetic fingerprints showed high similarity with the phylogenetic clustering, but had a much higher discriminatory power. However, whereas some correspondence between genotype and serotype could be observed, no relation could be found with virulence. In addition, similar to Vandenberghe et al. [56], a high phenotypic heterogeneity within the *V. anguillarum* isolates was observed. Nevertheless, again no correlation could be made with virulence. On the contrary, despite some exceptions, in general there was a good agreement between the phenotypic and genotypic clustering. One exception involves the two O3 strains VIB113 and CNEVA NB11008 which grouped in different clusters based on the phenotypic characterization, because of a few differences in carbon utilization. Strain VIB113 was for example unable to metabolize maltose and maltotriose in contrast to strain CNEVA NB11008. Furthermore, the distinct ecological niches from which these two strains were isolated, i.e. sea bass in France (CNEVA NB11008) versus rainbow trout in Denmark (VIB113), might explain the differences in carbon source utilization patterns. Keymer et al. [57] also observed a phenotypic diversity between coastal *Vibrio cholerae* strains isolated from different environments. An explanation for the low metabolic activity of VIB12 could not be found. This strain did not show any differences in growth characteristics in TSB +1% NaCl compared to the other *V. anguillarum* strains using the indirect Rapid Automated Bacterial Impedance Technique (RABIT) from Don Whitley Scientific (Shipley, UK) (data not shown), demonstrating similar growth kinetics among the different isolates.

In addition to the Omnilog Phenotyping, the production of extracellular enzymes potentially involved in virulence was determined for each isolate. Indeed, it was demonstrated that the avirulent strain VIB12 lacked both caseinase and hemolytic activity. However, no clear relation between enzymatic activity and virulence could be observed in this study. For example, although phospholipase activity has been previously described to be an important virulence factor for *V. vulnificus*, two virulent *V. anguillarum* strains, VIB103 and HI610, lacked phospholipase activity [58]. In addition, although strain VIB15 was identified as a virulent strain towards sea bass larvae, it was the only strain lacking lipase activity. Finally, no hemolytic activity was detected in avirulent strain 87-9-116, and in virulent strains 43, CNEVA NB11008, belonging to serotype O1, O2 and O3, respectively.

Altogether, the genetic and phenotypic properties of the 15 *V. anguillarum* isolates assessed in this study could not be correlated to the virulence towards sea bass larvae. This illustrates the complexity of the virulence mechanisms in *V. anguillarum* and suggests that virulence in *V. anguillarum* is highly multifactorial and cannot be assigned to one or a few crucial virulence factors. It is clear that further research is necessary to elucidate the underlying mechanisms of virulence differences in *V. anguillarum*. Using standardized virulence assays such as the one used in this study, together with whole genome sequencing [59], sequencing of whole transcriptomes [60] or epigenetics research [61], we should be able to increase our understanding of *V. anguillarum* virulence. However, anticipating on the different advantages of using a gnotobiotic screening method, it is acknowledged that real-life conditions will always be far more complex and that findings made under gnotobiotic conditions will need to be validated.

## Supporting Information

Table S1 Results of the OmniLog® PM Kinetic analysis of 15 selected Vibrio anguillarum strains grown on a PM1 Phenotype MicroArrayTM plate. For each strain and each carbon source, the area under the curve is presented. Values below 5000 and above 8000 are indicated in red and green respectively.
(XLS)
