# Correction: Does Virulence Assessment of *Vibrio anguillarum* Using Sea Bass (*Dicentrarchus labrax*) Larvae Correspond with Genotypic and Phenotypic Characterization?

**DOI:** 10.1371/annotation/c01fda12-3053-4443-815f-afedb3abd185

**Published:** 2013-10-15

**Authors:** Ingeborg Frans, Kristof Dierckens, Sam Crauwels, Ado Van Assche, Jørgen J. Leisner, Marianne H. Larsen, Chris W. Michiels, Kris A. Willems, Bart Lievens, Peter Bossier, Hans Rediers

The name of the fifth author was given incorrectly. The correct name is: Jørgen J. Leisner. 

The correct citation is: Frans I, Dierckens K, Crauwels S, Van Assche A, Leisner JJ, et al. (2013) Does Virulence Assessment of Vibrio anguillarum Using Sea Bass (*Dicentrarchus labrax*) Larvae Correspond with Genotypic and Phenotypic Characterization? PLoS ONE 8(8): e70477. doi:10.1371/journal.pone.0070477. 

The correct abbreviation in the Author Contributions Statement is: JJL. 

